# Impaired mitochondrial accumulation and Lewy pathology in neuron-specific FBXO7-deficient mice

**DOI:** 10.1186/s13041-022-00936-5

**Published:** 2022-06-14

**Authors:** Sachiko Noda, Shigeto Sato, Takahiro Fukuda, Shinichi Ueno, Norihiro Tada, Nobutaka Hattori

**Affiliations:** 1 Department of Neurology, Juntendo University Graduate School of Medicine, Tokyo, 113-8421 Japan; 2 Division of Neuropathology, Department of Neuropathology, The Jikei University School of Medicine, Tokyo, 105-8461 Japan; 3 Atopy Research Center, Juntendo University School of Medicine, Tokyo, 113-8421 Japan

**Keywords:** Parkinson’s disease, FBXO7, Dopaminergic neuron, Mitochondria, Synuclein, p62

## Abstract

Parkinson’s disease, the second most common neurodegenerative disorder, is characterized by the loss of nigrostriatal dopamine neurons. *FBXO7 (F-box protein only 7)* (PARK15) mutations cause early-onset Parkinson’s disease. FBXO7 is a subunit of the SCF (SKP1/cullin-1/F-box protein) E3 ubiquitin ligase complex, but its neuronal relevance and function have not been elucidated. To determine its function in neurons, we generated neuronal cell-specific FBXO7 conditional knockout mice (FBXO7^flox/flox^: Nestin-Cre) by crossing previously characterized FBXO7 floxed mice (FBXO7^flox/flox^) with Nestin-Cre mice (Nestin-Cre). The resultant Fbxo7^flox/flox^: Nestin-Cre mice showed juvenile motor dysfunction, including hindlimb defects and decreased numbers of dopaminergic neurons. Fragmented mitochondria were observed in dopaminergic and cortical neurons. Furthermore, p62- and synuclein-positive Lewy body-like aggregates were identified in neurons. Our findings highlight the unexpected role of the homeostatic level of p62, which is regulated by a non-autophagic system that includes the ubiquitin–proteasome system, in controlling intracellular inclusion body formation. These data indicate that the pathologic processes associated with the proteolytic and mitochondrial degradation systems play a crucial role in the pathogenesis of PD.

## Main text

Autosomal recessive mutations in the *FBXO7* (PARK15) gene are involved in a juvenile form of Parkinsonism with heterogenic phenotypes characterized by either a classic Parkinson’s disease (PD) phenotype, pyramidal tract signs only, or by a combination of Parkinsonism and pyramidal signs [1, 2]. FBXO7 is expressed in various types of tissues, including the gray and white matter of the brain [3].

**Fig. 1:**
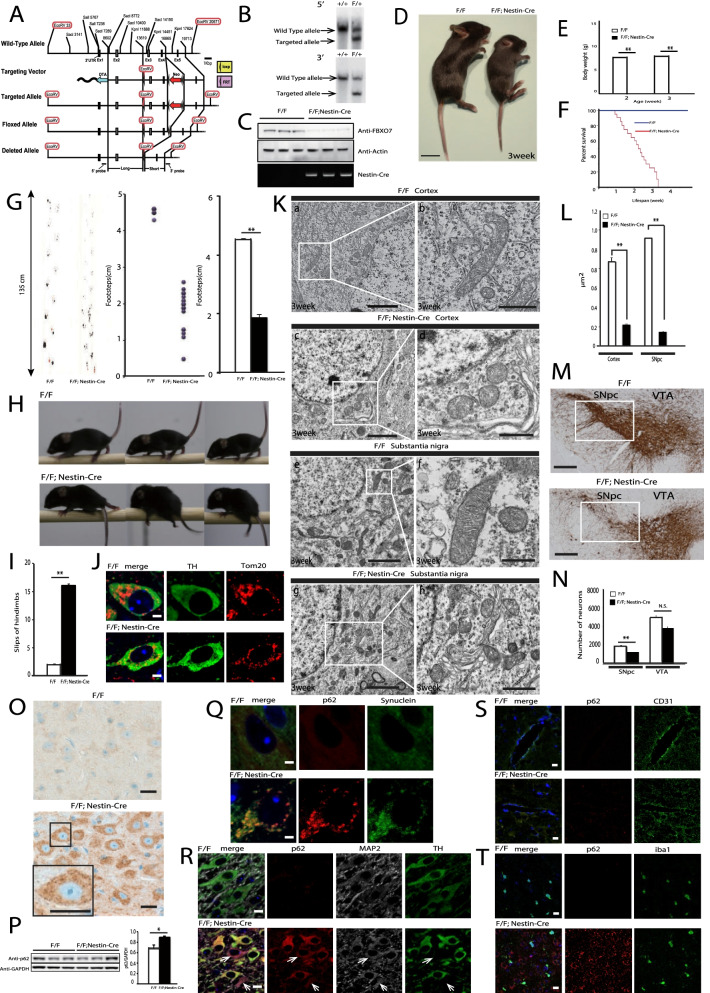
**A** Schematic representation of the targeting vector and the targeted allele of the *FBXO7* gene. The 3.2-kb region of the mouse *FBXO7* gene, including exons 3–4, was followed by an FRT-flanked PGK-neo expression cassette in the opposite transcriptional orientation. In the targeting construct, a 5.0-kb 5′ fragment and a 3.0-kb 3′ fragment were used as the long and short homologous arms, respectively. **B** Southern blot analysis of genomic DNA from ES cells that had undergone homologous recombination. Genomic DNA was digested with EcoRV and hybridized with the 5′ probe or 3′ probe. Upper panel: Bands detected by the 5′ probe. Lower panel: Bands detected by the 3′ probe. **C** Immunoblot of anti-FBXO7 antibody (Millipore #ABN1038) and actin (Millipore #MAB1501). Lanes 1–3: whole-brain tissues of 3-week-old FBXO7^flox/flox^ mice; lanes 4–6: whole-brain tissues of 3-week-old FBXO7^flox/flox^: Nestin-Cre mice. The genotypes of Nestin-Cre mice were determined by PCR using two primers: 5′-TTT GCC TGC ATT ACC GGT CGA TGC AAC-3′ and 5′-TGC CCC TGT TTC ACT ATC CAG GTT ACG GA-3′; these permitted the detection of the 1000-bp Nestin-Cre-targeted allele (lower panel). **D** 3-week-old FBXO7^flox/flox^ mice (left) and 3-week-old FBXO7^flox/flox^: Nestin-Cre mice (right). Scale bars: 1 cm. **E** Body weight of mice 2 or 3 weeks of age (2-week-old FBXO7^flox/flox^ mice and FBXO7^flox/flox^: Nestin-Cre mice, n = 10; 3-week-old FBXO7^flox/flox^ mice and FBXO7^flox/flox^: Nestin-Cre mice, n = 10). Data are presented as means ± SE (error bars); **p < 0.01 (significance was evaluated using Student’s t-test). **F** Kaplan–Meier analysis of survival of FBXO7^flox/flox^ mice (n = 20) and FBXO7^flox/flox^: Nestin-Cre mice (n = 20). **G** Footprint test in FBXO7^flox/flox^ mice and FBXO7^flox/flox^: Nestin-Cre mice. Red footsteps indicate forepaws; black footsteps indicate hindpaws. Each stride length was recorded. Data are means ± SE (error bars); **p < 0.01 (significance was evaluated using Student’s t-test). **H** Runway test of 3-week-old FBXO7^flox/flox^ mice (upper panel) and FBXO7^flox/flox^: Nestin-Cre mice (lower panel). The runway test was performed using a narrow, horizontally fixed beam. FBXO7^flox/flox^: Nestin-Cre mice could hardly move on the beam, and their hindpaws frequently slipped. **I** The number of hindlimb slips of mice crossing the 2-cm pole was recorded. Data are presented as means ± SE (3-week-old FBXO7^flox/flox^ mice and FBXO7^flox/flox^: Nestin-Cre mice, n = 10); data are means ± SE (error bars); **p < 0.01 (significance was evaluated using Student’s t-test). **J** Immunofluorescence labeling of Tom20 (red; Abcam #ab78547 Anti-Tom20) or TH (green; Merck Millipore #MAB318 Anti-Tyrosine Hydroxylase Antibody) in the SN area of FBXO7^flox/flox^ mice (upper panel) and FBXO7^flox/flox^: Nestin-Cre mice (lower panel). Scale bars: 2 μm. **K** For conventional electron microscopy, mice were fixed by cardiac perfusion with 2.5% glutaraldehyde in 0.1 mol/L phosphate buffer (pH 7.2). Brain slices were embedded in epoxy resin, and ultrathin sections (70-nm thickness) were prepared and imaged on an HT7700 electron microscope (Hitachi, Japan). Electron micrographs of the cerebral cortex (a, b, c, d) and dopaminergic neurons in the SN (e, f, g, h); 3-week-old FBXO7^flox/flox^ mice (n = 3) (a, b, e, f) and 3-week-old FBXO7^flox/flox^: Nestin-Cre mice (n = 3) (c, d, g, h). The right of each image (a, c, e, g) shows enlarged images. Scale bars: a, c, e, g, 2 µm; b, d, f, h: 500 nm. **L** Quantitation of mitochondrial area (dopaminergic and cerebral cortical cells from 3-week-old mice, n = 20). The mean mitochondrial area in dopaminergic neurons was smaller in 3-week-old FBXO7^flox/flox^: Nestin-Cre mice than in 3-week-old FBXO7^flox/flox^ mice. Significance was evaluated using Student’s *t*-test. *p < 0.01. **M** Histological analyses of the SN area in 3-week-old FBXO7^flox/flox^ mice (upper panel) and FBXO7^flox/flox^: Nestin-Cre mice (lower panel). Paraffin sections were immunostained for TH (Merck Millipore #MAB318 Anti-Tyrosine Hydroxylase Antibody) and are indicated by a square. Scale bars: 200 µm. **N** For stereological quantification, the ventral tegmental area (VTA) and substantia nigra pars compacta (SNpc) were selected. Every other 40-μm section of serial coronal brain slices for each genotype was stained for DAB. Quantification was performed with a design-based stereology system (Stereo-Investigator version 2020; MBF Bioscience, Williston, VT, USA). The sampling parameters were defined according to the software guide to achieve a coefficient of error ranging from 0.05 to 0.09 as determined using the Gundersen test. Data are means ± SE (3-week-old FBXO7^flox/flox^ mice, n = 3; 3-week-old FBXO7^flox/flox^: Nestin-Cre mice, n = 3); **p < 0.01 (Student’s *t*-test). N.S.: Not significant. **O** Histological analyses of p62 (PROGEN #GP62-C Anti-p62/SQSTM1) in 3-week-old FBXO7^flox/flox^ mice (upper panel) and FBXO7^flox/flox^: Nestin-Cre mice (lower panel). The area in the small rectangle is enlarged in the inset image at the lower left. Scale bars: 10 µm. **P** Immunoblot for p62 (PROGEN #GP62-C Anti-p62/SQSTM1) and GAPDH (Proteintech #10494-1-AP). Lanes 1–3: 3-week-old FBXO7^flox/flox^ mice; lanes 4–6: whole-brain tissues of 3-week-old FBXO7^flox/flox^: Nestin-Cre mice. Data are means ± SE (3-week-old FBXO7^flox/flox^ mice, n = 3; 3-week-old FBXO7^flox/flox^: Nestin-Cre mice, n = 3); *p < 0.05 (Student’s *t*-test). **Q** Immunofluorescence labeling of p62 (red; PROGEN #GP62-C Anti-p62/SQSTM1) or Synuclein (green; Merck Millipore #AB5038P Anti-Synuclein Alpha Antibody) in the SN area of FBXO7^flox/flox^ mice (upper panel) and FBXO7^flox/flox^: Nestin-Cre mice (lower panel). Scale bars: 2 μm. **R** Immunofluorescence labeling of p62 (red; PROGEN #GP62-C Anti-p62/SQSTM1), MAP2 (gray; Genetex #GTX11267 Anti-MAP2), or TH (green; Merck Millipore #MAB318 Anti-Tyrosine Hydroxylase Antibody) in the SN area of FBXO7^flox/flox^ mice (upper panel) and FBXO7^flox/flox^: Nestin-Cre mice (lower panel). Arrows indicate interneurons. Scale bars: 5 μm. **S** Immunofluorescence labeling of p62 (red; PROGEN #GP62-C Anti-p62/SQSTM1) or CD31 (green; R&D #AF3628 Anti-CD31) in the SN area of FBXO7^flox/flox^ mice (upper panel) and FBXO7^flox/flox^: Nestin-Cre mice (lower panel). Scale bars: 10 μm. **T** Immunofluorescence labeling of p62 (red; PROGEN #GP62-C Anti-p62/SQSTM1) or iba1 (green; Wako #NCNP24 iba1 Monoclonal Antibody) in the SN area of FBXO7^flox/flox^ mice (upper panel) and FBXO7^flox/flox^: Nestin-Cre mice (lower panel). Scale bars: 10 μm

To determine the function of FBXO7 in vivo, we generated FBXO7^flox/flox^ mice. A targeting vector was constructed using 5.0- and 3-kb DNA fragments as the 5′ and 3′ homologous sequences, respectively (Fig. 1A). The linearized targeting vector was transfected into C57BL/6 embryonic stem (ES) cells. Selected clones were screened for homologous recombination by Southern blotting. Using the 5′ external probe and a probe specific for the neo sequence, we confirmed that the clones carried the desired homologous recombination. ES cells derived from these clones were injected into C57BL/6 embryos. Chimeric offspring were crossed with C57BL/6 mice to obtain germline transmission, which was confirmed by Southern blot analysis with the 5′ (Fig. 1B upper panel) and 3′ (Fig. 1B lower panel) probes. Heterozygous mice were then interbred to obtain homozygous knockout and wild-type control mice. All animals were kept in a pathogen- and odor-free environment, which was maintained under a 12-h light/dark cycle at ambient temperature. Procedures were approved by the Animal Experimental Committee of the Juntendo University Graduate School of Medicine and performed in accordance with the guidelines of the National Institutes of Health and the Juntendo University Graduate School of Medicine. Next, we generated neuronal cell-specific FBXO7^flox/flox^: Nestin-Cre mice by crossing the previously characterized FBXO7^flox/flox^ mice with FBXO7^flox/flox^: Nestin-Cre mice harboring the Cre recombinase-coding sequence downstream of a characterized fragment of the Nestin promoter, and confirmed that FBXO7 protein levels were decreased in the whole brain of Cre-expressing mice (Fig. 1C).

FBOX7^flox/flox^: Nestin-Cre mice were viable at birth and indistinguishable in appearance from their littermates, but experienced gradually increasing weight loss (Fig. 1D, E) and had a markedly lower survival rate after birth (Fig. 1F). FBXO7^flox/flox^: Nestin-Cre mice began to show impairment in motor coordination tasks and motor behavioral deficits, as determined by the footprint test (Fig. 1G) and runway test (Fig. 1H). We first conducted the footprint test, because a short stride is a characteristic of PD, including in patients with PARK15 mutations. FBXO7^flox/flox^: Nestin-Cre mice had a shorter stride than FBXO7^flox/flox^ mice (Fig. 1G). Regarding the runway test, while FBXO7^flox/flox^ mice exhibited well-coordinated movement and almost no slips of the forepaw or hindpaw from the beam, FBXO7^flox/flox^: Nestin-Cre mice could hardly move on the beam and slipped frequently (Fig. 1H). In particular, the hindpaws of FBXO7^flox/flox^: Nestin-Cre mice often slipped off the beam (Fig. 1I). Gait disturbance progressed, and by the terminal stage, the majority of affected mice could hardly move.

Mitochondrial damage and dysfunction in the substantia nigra have been previously reported in patients with sporadic PD [4, 5]. These observations support the notion that aberrant mitochondrial function is a critical contributor to pathological neuronal degeneration. Accumulating knowledge regarding PINK1 and Parkin, both of which are associated with mitochondria, has increased our understanding of mitochondrial quality control [6, 7].

FBXO7 and Parkin are interaction partners. FBXO7 is recruited to damaged mitochondria, and is required for the successful recruitment of Parkin. FBXO7 also binds to PINK1, and PINK1, Parkin, and FBXO7 act in concert to control the events leading to mitophagy. Interestingly, the expression of human FBXO7 in a Parkin mutant fly model rescues its phenotype, which is characterized by mitochondrial disruption and locomotor defects [8].

To characterize the damaged mitochondria in FBXO7^flox/flox^: Nestin-Cre mice, we performed immunohistological (Fig. 1J) and ultrastructural (Fig. 1K) analysis of cortical and dopaminergic neurons of 3-week-old mice. We observed small, round, fragmented mitochondria in these neurons in FBXO7^flox/flox^: Nestin-Cre mice but not in FBXO7^flox/flox^ mice. Precise quantification revealed that the mitochondrial area was reduced in cortical and dopaminergic cells (Fig. 1L). Together, these observations suggest that mitochondrial fragmentation might be facilitated in FBXO7-deficient mice. In order for damaged mitochondria to be degraded by autophagy, they must be segregated by fission [9]. Our in vivo results are reasonable if FBXO7-mediated mitophagy contributes to mitochondrial degradation systems.

To assess the pathological contribution of damaged mitochondria, we compared tyrosine hydroxylase (TH)-immunoreactive neurons between FBXO7^flox/flox^: Nestin-Cre and control mice. As demonstrated in the runway test (Fig. 1H, I), FBXO7^flox/flox^: Nestin-Cre mice exhibited locomotor dysfunction. These FBXO7^flox/flox^: Nestin-Cre mice had fewer TH neurons in the central portion of the substantia nigra (SN) pars compacta (Fig. 1M, N), where the reduction in TH cell number was most prominent in other PD model mice [3, 10–13].

Finally, to identify the relationship between our model mice and PD, we conducted immunohistological and blot analysis in the brain. Interestingly, in FBXO7^flox/flox^: Nestin-Cre mice we identified p62-positive aggregates (Fig. 1O) that exhibited a significant increase in p62 levels (Fig. 1P) and synuclein colocalization (Fig. 1Q). These pathologies were identified in the dopaminergic neurons (Fig. 1R), including internal neurons (Fig. 1R arrows). Because p62 aggregates were not seen in endothelial cells (Fig. 1S) or microglia (Fig. 1T), FBXO7^flox/flox^: Nestin-Cre mice were thought to demonstrate a neuron-specific phenotype. Further research is needed, but our results suggest that loss of FBXO7 may affect p62 and synuclein proteolysis, and impaired mitochondria elimination.

## Data Availability

All data generated or analyzed during this study are included in this published article.

## Abbreviations

PD: Parkinson’s disease
EO-PD: Early-onset Parkinson’s disease
FBXO7: F-box protein only 7
UPS: Ubiquitin–proteasome system
TH: Tyrosine hydroxylase
SN: Substantia nigra
ES: Embryonic stem
VTA: Ventral tegmental area
SNpc: Substantia nigra pars compacta
